# Chit-Chat

**Published:** 1874-07

**Authors:** 


### Editorial Chit-Chat.

A Beautiful Roe.—The block of so-call- buildings on the upper end of the burnt district. What a blessing to the city enterprising men are.

All Aboard.—The Bessemer anti-seasick saloon steamer is finished and ready to receive passengers. Persons with weak stomachs, and great desire to go abroad, will rejoice thereat.

Give it Up.—And this is what our managerial friend propounded: Are blacksmiths, who make a livilihood by forging, or carpenters, who occasionally do counter-fitting, any worse than men who sell iron and steel for a living ?

Cataract.—We have nearly completed our fourth hundred of operations for *cataract*. We think we can show a larger proportion of cures than any similar institution in the country. We are preparing a report of the cases, which we hope soon to publish.

A Shot.—Rev. Thomas K. Beecher can hit a pint bottle, with his rifle, at 80 yds., and knock it into flinders. We have seen him do it repeatedly. He and Dr. Gleason are splendid shots with a rifle, and amuse themselves in their leisure hours in killing wood-chucks, at long range.

The Gazette.—We learn that Mr. S. C. Taber and Col. DeVoe have purchased this enterprising paper. This assures the *Gazette* of renewed success and prosperity. Charles Hazard remains as "ye local," than whom a better one does not exist. With Sam and Charley the *Gazette* will sparkle with wit and wisdom.

Why Is It ?—We want to know; it has always puzzled us, how it is that girls can always tell a married man from a single one ! They can do it, we have caught them at it again and again. Only recently, we stopped at a farmhouse for our accustomed bowl of bread-and-milk. We were angling for trout, and of course, were tidily attired. The mother asked the daughter—a young and buxom lass—to bring us the required article of diet. Stepping outside the door, we were grieved to hear that fair damsel inform her mother, *sotto voce*, that she might go herself, saying, "he is only an old married cuss." Again we repeat, how did she know ?

CREMATION.—Extremes meet. We have always had to earn our living, and now they want to compel us to urn our dead.

QUEER.—We have just received information of the death of an acquaintance, who suddenly expired while engaged in the act of tying his neck-tie. Why will young men persist in this dangerous habit? We have not worn a neck-tie for years.

NOTE IT.—The new medical society recently formed in this city, includes ladies—those who are regular graduates in medicine, as well as gentlemen. This is the first instance of the kind that has ever occurred in the Southern Tier.

NEW MEDICAL SOCIETY.—There has been a new medical society formed in this city comprised of gentlemen too liberal in their views to be classed with the regulars. They have joined the "Eclectic Medical Society of the State of New York."

ACADEMY PRIZE.—Among the many prizes carried from the Academy, the most valuable one was in the possession of Miss Mame Cooley, eldest daughter of Jesse Cooley, Esq. It consisted of an elegant gold watch and chain, of elaborate style and finish, a present from father to daughter, upon her graduation day. The present was elegant, so was the young lady, therefore, both worthy of each other.

OUR MEDICAL ITEMS AND NEWS.—We call attention of physicians to the excellence of this department of the BISTOURY. We expend much time and labor upon it, and think we present a better budget of medical items, and of more real value to the practicing physician, than any other medical journal of our acquaintance. This feature of the BISTOURY alone, is worth many times more than the subscription price.

FOUND AT LAST.—We know of a young man who has an eye single to business success, and was studying for the ministry in one of the one hundred and eight theological schools of the United States. Before the completion of his first year he discovered that this is one of the worst paid professions, as people in general care more for their bodies than for their souls; so at the nick of time, he changed his mind and studied medicine in one of the hundred medical colleges of the land. When almost through this very useful course of training, he discovered another important fact, namely, that people care more for their pockets than for their bodies. This settled the matter definitely; he changed his profession again, and is succeeding admirably as a lawyer.

OUR SCHOOLS.—We are glad to note the growing interest manifested by parents in our public schools. At the recent Commencement exercises the school rooms were crowded with delighted listeners. This is an encouragement offered teachers and pupils that will redound to the profit of all concerned. We were sorry that our time would only permit a visit to the Academy and Grammar School No. 3. We were delighted with what we saw and heard, particularly in No. 3. Mr. Coburn and assistants exhibited a well trained and disciplined lot of scholars that did them much credit. The exercises were remarkably interesting, the pupils acquitting themselves with very marked ability.

THAT THUNDER STORM.—In our recent camp life we had the pleasure of witnessing a magnificent pyrotechnic display in the heavens, at mid-night, while we were sheltered only with a common field tent. The storm was fearfully grand, the flashes of lightning dazzlingly bright and vivid, while the thunder seemed to lash the mountains until the entire valley trembled with its terrible roar. We say we enjoyed it—the ex-banker and I—but not so with our ex-merchant and manager friend. The first buried himself as deeply as possible in the straw, holding a pillow over his face, while the latter converted a large Saratoga trunk into a novel hiding place for a duly frightened and nearly smothered manager. In the morning his whereabouts was discovered by an inquiry coming through the key-hole as to whether the storm had subsided.

SURGICAL NOVELTIES.—Great novelties are constantly being constructed by scientific surgeons in all parts of the world. The most recent are the inventions of Dr. P. Smith, of the "Aspirator," which has been so extensively and usefully employed by Dr. Dieulafoy, of Paris. By this instrument fluids can be removed from deeply seated cavities, through a very small puncture, with great certainty and safety. Matter can be removed from the lungs by puncturing the chest between the ribs, almost painlessly, and medicines are introduced through the same opening.

Prof. Esmarch has introduced a plan by which amputations can be performed without loosing one drop of blood. This is accomplished by applying an elastic bandage, made of rubber webbing, winding it from the toes or fingers up the leg or arm to a point above where the amputation is to be made. An elastic cord is then placed about the limb, compressing both arteries and veins, so that no blood remains in that portion of the limb to be removed. This renders the amputation absolutely bloodless.

---

THE HOBOKEN TURTLE CLUB.—This is a band of gentlemen, residing in New York, who pride themselves upon their gastronomic capabilities, especially when applied to the green turtle. They enticed some of our Elmira epicures within reach of the flavor of their favorite reptile, the result being the formation of the "Elmira Turtle Club," with his Honor, Major Luther Caldwell, as chief turtle in the puddle. The latter gave a reception to the former, in this city recently, when Maxey Haight supplied turtle soup literally by the barrel. If we thought our abdomen would swell to the proportion of that of every individual member's of the Hoboken club, by simply imbibing turtle soup, (and they assured us such would be the result,) we would feel inclined to join the Major's turtles in this city. The dinner was a good one—turtle being the sole bill of fare, and all parties seemed to have a jolly good time.

---

THE BOYS.—Our second son has just startled us by dropping a box by our side, upon which he has seated himself, preparatory to wiping the sweat from his brow, induced by the load which he has been engaged in carrying. To our inquiry as to what the box contained, we gleaned the following facts : "What's them? why, them's fire-crackers,—next Saturday's the fourth, didn't you know it ?"

But, my son, how many crackers have you there ? "Only forty packs, me and Thad bought 'em for three dollars, part of 'em's mine, and part his,—jolly, won't we have lots 'o fun shootin' 'em, though ?

We coincided in his opinion, but secretly resolved to take up our station in the observatory of the Institute, for the major portion of "the day we celebrate." The boys are entitled to a "good time" on the fourth—we are glad to see them enjoy it ; and, instead of attempting to suppress their pent up patriotism which will explode once a year, we should give them unlimited liberty for one day, and let them "have it out." When you become tired of the din, *re-tire*, as we do, and let the boys go it.

---

WOULD'NT SET.—A friend sent us some mocking-bird eggs, neatly packed, from the forests of Florida. Our instructions were to place them under a robin, when we would likely procure mocking-birds properly acclimated, and would do better than those imported from the South. We obeyed instructions, watching a pair of busy robins, until their habitation had been properly prepared, and their eggs duly laid. Climbing the tree with our mocking-bird eggs, we placed them tenderly in the nest, extracting an equal number of the robin's eggs. Satisfied with our experiment so far, we descended and awaited results ; and this is what we saw: Mrs. Robin returned to the nest, and, perching herself on its side quizzically contemplated the new eggs, first from one eye, then from the other. She turned them over with her bill and examined the other side of them, they being of a dirty blue color, did not exactly correspond to her own, and she probably thought they were somewhat soiled, and desired to see how they looked on the other side. Poking them about for a while and scrutinizing them as before, she became uncertain as to their identity and flew away. Presently she returned with Mr. Robin and introduced him to the new lay. Perched upon the side of the nest, he looked from one eye upon the eggs, poked them with his bill, looked again, and then took up his position by the side of his mate, when, if ever there was a consultation held, it was done there. They chatted earnestly together, both examined the eggs again, and Mrs. Robin took her seat upon the nest, while Mr. Robin shut one eye and squinted at us with the other, and then flew away, saying something to his mate as he went. Mrs. Robin occupied the nest five minutes, got up, surveyed the eggs once more, took a suspicious look at us, gave a comical wink, as much as to say, "no you don't," flew away after her mate, and in ten minutes returned with him, both with their bills full of straw, and immediately commenced the erection of a new homestead.—Those two robins are evidently disbelievers in the propriety of miscegenation.

